# Activation of the clock gene *TIMELESS* by H3k27 acetylation promotes colorectal cancer tumorigenesis by binding to Myosin-9

**DOI:** 10.1186/s13046-021-01936-4

**Published:** 2021-05-10

**Authors:** Meng Cao, Yi Wang, Yijing Xiao, Dandan Zheng, Chunchun Zhi, Xin Xia, Xiaoqin Yuan

**Affiliations:** 1 Department of General Surgery, Nanjing Drum Tower Hospital, the Affiliated Hospital of Nanjing University Medical School, Nanjing, 210008 China; 2 Department of Anatomy, Histology and Embryology, Nanjing Medical University, Xuehai Building, Rm D509, 101 Longmian Avenue, Jiangning District, Nanjing, 211166 China; 3 Jiangsu Key Laboratory of Oral Diseases, Nanjing Medical University, Nanjing, 210029 China; 4 Department of Pathology, Nanjing Jinling Hospital, Nanjing University School of Medicine, Nanjing, 210002 China; 5 Key Laboratory for Aging & Disease, Nanjing Medical University, Nanjing, 211166 China

**Keywords:** Timeless, Myosin-9, Wnt/β-catenin pathway, Colorectal cancer

## Abstract

**Background:**

Colorectal cancer (CRC) is a common tumor characterized by its high mortality. However, the underlying molecular mechanisms that drive CRC tumorigenesis are unclear. Clock genes have important roles in tumor development. In the present study, the expression and functions of clock gene *TIMELESS* (encoding the Timeless protein) in CRC were investigated.

**Methods:**

Immunohistochemistry, cell proliferation, migration, invasion, EMT and xenograft tumor experiments were used to prove the function of Timeless in the tumorigenesis of CRC. Immunoprecipitation, mass spectrometry, Immunofluorescence and Chromatin immunoprecipitation (ChIP) were utilized to clarify the mechanism of Timeless in regulating CRC tumorigenesis.

**Results:**

We found that Timeless was upregulated in CRC tissues compared with corresponding normal tissues and its expression was closely associated with the TNM stages and overall survival of CRC patients. Functional studies demonstrated that Timeless promoted the proliferation, invasion, and EMT of CRC cells in vitro and in vivo. Mechanistic investigations showed that Timeless activated the β-catenin signal pathway by binding to Myosin-9, which binds to β-catenin to induce its nuclear translocation. The upregulation of Timeless was attributed to CREB-binding protein (CBP)/p300-mediated H3K27 acetylation of the promoter region of Timeless.

**Conclusion:**

Timeless regulates the tumorigenesis of CRC by binding to and regulating myosin-9, suggesting Timeless might be a potential prognostic biomarker and therapeutic target for CRC.

## Background

Colorectal cancer (CRC) is a common tumor worldwide with a high mortality rate [1]. With an incidence ranking the third among all cancers, CRC has been proven as the most common cause of cancer death in the United States [2]. In China, CRC is a commonly-diagnosed cancer with 521,490 cases (12.2% of total cancer cases) and 247,563 cancer-related deaths (8% of total cancer deaths) reported in 2018 [3]. However, the underlying molecular mechanisms that drive CRC tumorigenesis are poorly understood.

Accumulating evidence suggests that the dysregulation of circadian clock gene expression including *CLOCK1, CRY1, CRY2,* and *PER1–3* is associated with the increased incidence and metastasis of cancers such as colorectal cancer, breast cancer, and glioma [4–8]. Perturbations of circadian clock genes impact key pathways affecting cancer development and progression, including cell-cycle control, apoptosis, metabolic regulation, epithelial-to-mesenchymal transition, immunity, and DNA damage responses [8–11].

*TIMELESS* (*TIM*), an evolutionarily-conserved circadian gene encoding the Timeless protein, interacts with Cryptochrome (CRY) and Period (PER) proteins and acts on the negative arm of the circadian cycle [12–15]. Timeless, as a replication fork-associated factor, interacts with Timeless-interacting protein (Tipin) to form a stable complex that affects replication checkpoints and normal DNA replication [16, 17]. Timeless also interacts with PARP-1 to promote DNA-damage repair [18, 19].

Recently, Timeless has been reported to be upregulated in various types of cancer and to be involved in cancer development, progression, and associated with poor patient survival. For example, in breast cancer, Timeless was significantly upregulated in tumor tissues and patients with high Timeless levels had a poorer prognosis than patients with low Timeless levels [20]. Timeless overexpression was associated with poor clinical survival and lymph node metastasis in early-stage cervical carcinoma [21]. Moreover, Timeless was an independent prognostic factor for overall survival and progression-free survival in nasopharyngeal carcinoma [22] and CRC tissues had higher levels of Timeless mRNA compared with the corresponding healthy mucosa of CRC patients. In addition, high Timeless mRNA levels were associated with TNM stages III-IV and microsatellite instability [4]. However, the precise role of Timeless in the progression of CRC is poorly understood.

In this study, we aims to investigate the expression of Timeless in CRC tissues and demonstrate the function and mechanism of Timeless in the proliferation, migration, invasion and EMT of CRC cells. Our results may provide a diagnostic and prognostic biomarker of CRC.

## Materials and methods

### Clinical samples

A total of 114 pairs of paraffin-embedded human CRC specimens and corresponding adjacent normal mucosa samples were histopathologically diagnosed at the First Affiliated Hospital of Nanjing Medical University and Nanjing Drum Tower Hospital. The detailed clinicopathologic characteristics of the CRC patients are summarized in Table 1. This project was approved by the Research Ethics Committee of Nanjing Medical University (Approval ID: (2016)640) and Research Ethics Committee of Nanjing Drum Tower Hospital (Approval ID: 2020–378-02).

**Table 1:** Association of Timeless expression with clinicopathological variables in patients with CRC (*n* = 114)

Variable	Group	Number of cases (%)	Timeless expression		*P* value
			High	Low	
Gender					
	male	66 (57.9)	33	33	1
	female	48 (42.1)	24	24	
Age (years)					
	≥60	70 (61.4)	41	29	0.021*
	<60	44 (38.6)	16	28	
Tumor Diameter (cm)					
	> 5	43 (37.7)	19	24	0.334
	≤5	71 (62.3)	38	33	
Tumor Location					
	Colon	76 (66.7)	39	37	0.691
	Rectum	38 (33.3)	18	20	
Clinical stage					
	I-II	55 (48.2)	18	37	0.000***
	III-IV	59 (51.8)	39	20	
T stage					
	T1–2	20 (17.5)	5	15	0.014*
	T3–4	94 (82.5)	52	42	
N stage					
	absent	57 (50)	17	40	0.000***
	present	57 (50)	40	17	
M stage					
	M0	100 (87.7)	47	53	0.087
	M1	14 (12.3)	10	4	
Vascular invasion					
	absent	85 (74.6)	36	49	0.005**
	present	29 (25.4)	21	8	
Perineural invasion					
	absent	71 (62.3)	34	37	0.562
	present	43 (37.7)	23	20	

* *P* < 0.05, ** *P* < 0.001, *** *P* < 0.0001

### Immunohistochemistry (IHC) staining and evaluation

IHC staining was performed on paraffin-embedded tissues using anti-Timeless antibody (Abcam, Cambridge, UK) according to a method described previously [23]. IHC results were scored using the modified Histoscore (H-score) [24]. Briefly, in five fields at × 400 magnification chosen randomly, the staining intensity of cell nuclei was scored as 0, 1, 2 or 3, corresponding to negative, weak, moderate and strong staining, respectively. The total number of cells in each field and the number of cells stained at each intensity were counted. This semiquantitative assessment was performed by two independent investigators blinded to the clinical parameters of the corresponding patients. The H-score was determined according to the following formula: [(% of weak staining) × 1] + [(% of moderate staining) × 2] + [(% of strong staining) × 3], yielding a range from 0 to 300 [25]. The expression level of each protein was categorized as low or high according to the median H-score.

### Cell culture

The human CRC cell lines HCT116, RKO, HT29, CX-1, SW620, Caco-2, and LoVo were purchased from the Shanghai Institute of Cell Biology, Chinese Academy of Sciences. HCT116, RKO, CX-1, SW620, and LoVo cells were cultured in RPMI 1640 medium (HyClone, South Logan, UT, USA), and other cell lines were maintained in DMEM medium (HyClone), supplemented with 10% FBS (Gibco, Grand Island, NY, USA), 100 U/mL penicillin, and 0.1 mg/mL streptomycin (Gibco). All cell lines were cultured at 37 °C in a humidified incubator of 5% CO_2_. The cells were collected for further studies after treatment with cycloheximide (CHX) (MedchemExpress, Monmouth Junction, NJ, USA), C646 (MedchemExpress), or MG132 (Sigma-Aldrich, St. Louis, MO, USA) for different periods.

### Small interfering RNA and plasmid transfection

Timeless, Myosin-9, P300 and CBP were knocked down by small interfering RNAs (siRNA) targeting Timeless (si-TIM), Myosin-9 (si-MYH9), P300 (si-P300) and CBP (si-CBP) (RiboBio, Guangzhou, China). Their scramble controls (si-NC) (RiboBio) were transfected into CRC cells with Lipofectamine RNAiMAX (Invitrogen, Waltham, MA, USA). Timeless and Myosin-9 cDNA were cloned respectively into pcDNA3.1 expression vectors to construct pcDNA-TIM and pcDNA-MYH9, respectively. A plasmid expressing β-catenin (pcDNA-Ctnnb1) was a gift from Dr. Yuping Lai (East China Normal University). The plasmids were transfected into CRC cells using Lipofectamine 2000 (Invitrogen) according to the manufacturer’s protocol. The siRNA sequences for Timeless, Myosin-9, and CBP are as follows:

TIM siRNA1: sense: 5′-GUAGCUUAGCCUUUCAAATT-3′, antisense: 5′-UUUGAAAGGACUAAGCUACTT-3′; siRNA2: sense: 5′-AGAAGAGAAGGAAGAAGAATT-3′, antisense: 5′-UUCUUCUUCCUUCUCUUCUTT-3′; and siRNA3: sense: 5′-GCCUACAUGUGCUAGAGAUTT-3′, antisense: 5′-AUCUCUAGCACAUGUAGGCTT-3′. MYH9 siRNA: sense: 5′-GGAGCGGAACACUGACCAGTT-3′, antisense: 5′-CUGGUCAGUGUUCCGCUCCTT-3′CBP siRNA: sense: 5′-GGAGCCAUCUAGUGCAUAATT-3′ antisense: 5′-UUAUGCACUAGAUGGCUCCTT-3′; p300 siRNA: sense: 5′-AACCCCUCCUCUUCAGCACCATT-3′, antisense: 5′-UGGUGCUGAAGAGGAGGGGUUTT-3′.

### Generation of stable Timeless knockdown cells

A lentivirus harboring sh-TIM was constructed by Genechem (Shanghai, China), and HCT116 cells were infected with an MOI of 20. After infection, 2 μg/ml puromycin (Sigma) was used to select stably transduced cells. HCT116 cells infected with lentivirus expressing scrambled shRNA with puromycin resistance were used as controls. The scrambled shRNA sequence and the shRNA targeting Timeless were TTCTCCGAACGTGTCACGT and GAAGGATCTGATCCGCTAT, respectively.

### CCK8 assay

Cell growth was assessed using the Cell Counting Kit 8 (MedchemExpress). In brief, 3000 cells transfected with siRNA or plasmid were seeded into 96-well plates per well in 100 μl culture medium. Cell growth was determined at 0, 24, 48, 72, and 96 h following the manufacturer’s instructions. The absorbance at a wavelength of 450 nm was measured in a microplate reader (Bio-Tek, Winooski, VT, USA).

### Colony formation assay

The cells transfected with siRNA or plasmid were seeded into 6-well plates (1000 cells/well) and cultured in medium containing 10% FBS for 10 to 15 days, The medium was replaced every 3 days. Colonies were fixed with 4% formaldehyde for 15 min and stained with 0.1% crystal violet solution (Keygene, Nanjing, China) for 15 min. The crystal violet stained colonies were counted to determine colony formation.

### Transwell assays

Cell migration and invasion assays were performed with 8-μm pore Transwell chambers (Corning, NY, USA) as previously described [23]. Overall, 5 × 10^4^ or 8 × 10^4^ cells in 200 μl serum-free medium were seeded in the upper chamber coated with or without Matrigel for migration or invasion assays, respectively. The lower chambers were filled with 600 μL medium with 10% FBS for migration or with 20% FBS for invasion. After 24–36 h, the cells on the bottom surface of the membrane were fixed with 95% ethanol and stained with 0.1% crystal violet. Five random fields (× 200) were counted using a microscope to assess cell migration and invasion.

### Tumor formation and metastasis assays in nude mouse models

Male nude BALB/c mice (4–5 weeks old) were purchased from the Model Animal Research Center of Nanjing University (Nanjing, China) and maintained on a 12-h light/12-h dark cycle under pathogen-free conditions. For xenograft animal models, the mice were randomly divided into two groups, each consisting of five mice. After anesthetization, 5 × 10^6^ Timeless-knockdown stable HCT116 cells or control cells were injected subcutaneously into the flank of mice. The size of tumors was measured twice a week for 4 weeks, and the tumor volume was calculated according to the equation: volume = length × width^2^ × 0.5. After 4 weeks, all mice were sacrificed and the xenografts were dissected and weighed. For the in vivo lung metastasis assay, Timeless-knockdown stable HCT116 cells and control cells (4 × 10^6^) were injected into the tail vein of each nude mouse (*n* = 5 for each group). These mice were sacrificed after 10 weeks and examined for lung metastases. Animal welfare and experimental procedures were approved by the Institutional Animal Care and Use Committee of Nanjing Medical University (Approval ID 1601080). All protocols were performed in accordance with the Guide for the Care and Use of Laboratory Animals.

### Western blot analysis

Protein lysates from cells or tumor tissues were separated by sodium dodecyl sulfate polyacrylamide gel electrophoresis (SDS-PAGE), transferred onto polyvinylidene fluoride (PVDF) membranes, and blotted using the following antibodies: Timeless (Abcam, Cambridge, UK), Myosin-9 (Abcam), Snail (Abcam), E-cadherin (CST, Danvers, MA, USA), N-cadherin (Abcam), β-catenin (Abcam), Histone H3 (CST), H3K27ac (Abcam), CBP (Santa Cruz Biotechnology, CA, USA), P300 (Abcam), GAPDH (Santa Cruz Biotechnology), and Flag (Sigma-Aldrich).

### Immunoprecipitation (IP) and mass spectrometry

HCT116 cells were transfected with Flag-tagged pcDNA-TIM, pcDNA-MYH9, or pcDNA-Ctnnb1, and pcDNA3.1 was used as a negative control. After transfection, the cells were lysed with RIPA lysis buffer. Then, cell lysates were incubated with anti-Flag magnetic beads (Sigma-Aldrich) overnight at 4 °C. After incubation, the beads were washed with lysis buffer. The precipitated proteins were eluted with SDS sample buffer and analyzed by Western blotting with anti-Flag (Sigma), anti-Myosin-9 (Abcam), anti-Timeless (Abcam), and anti-β-catenin (Abcam) antibodies.

To identify proteins that bind to Timeless, we immunoprecipitated cell lysates with Timeless antibody (Abcam) or IgG. Then, the proteins precipitated by Timeless antibody and IgG were detected by mass spectrometry analyses (Shanghai Applied Protein Tech, Shanghai, China).

### RNA extraction and real-time RT-PCR

Total RNA was extracted from cells with TRIzol reagent (Invitrogen, Grand Island, NY, USA). RT-qPCR was carried out with a PrimeScript™ 1st Strand cDNA Synthesis Kit (Takara Bio, Shiga, Japan) and SYBR green reagents (Takara Bio) according to the user’s manual and detected in an ABI 7300 sequence detector (Applied Biosystems, Foster City, CA, USA). ACTIN was used for normalization. The PCR primers used in this study were: ACTIN-F: 5′-TCATGAAGTGTGACGTGGACAT-3′, ACTIN-R: 5′-CTCAGGAGGAGCAATGATCTTG-3′; TIM-F: 5′-CACCAGGACAAGCCTCTCTTT-3′, TIM-R: 5′-TTAGGCAGATTGCCAAAACAGA-3′; CBP-F: 5′-CGGCTCTAGTATCAACCCAGG-3′, CBP-R: 5′-TTTTGTGCTTGCGGATTCAGT-3′;p300-F: 5′-AATCCTTTCCATACCGAACC-3′, p300-R: 5′-GAGGGCAGTCAGAGCCATAC-3′; MYH9-F: 5′-CCTCAAGGAGCGTTACTACTCA-3′, MYH9-R: 5′-CTGTAGGCGGTGTCTGTGAT-3′; AXIN2-F: 5′-GGTTTCCCCTTGGACCTCG-3′, AXIN2-R: 5′-CCGTCGAAGTCTCACCTTTAATG-3′; CCND1-F: 5′-GCTGCGAAGTGGAAACCATC-3′, CCND1-R: 5′-CCTCCTTCTGCACACATTTGAA-3′; LEF1-F: 5′-GAGAAAAGTGCTCGTCACTGT-3′, LEF1-R: 5′-TGCCAAATATGAATAACGACCCA-3′.

### Nuclear and cytoplasmic protein extraction

Nuclear and cytoplasmic proteins were extracted using a Kit (Beyotime Biotechnology, Shanghai, China) following the manufacturer’s instructions. All samples were stored at − 80 °C for further use.

### Immunofluorescence

The cells were seeded on coverslips in 24-well plates and cultured for 24 h. Then, the cells were fixed with 4% paraformaldehyde for 15 min, permeabilized with 0.2% Triton X-100 in PBS, blocked with 5% bovine serum albumin (BSA), followed by incubation with specific primary antibodies at 4 °C overnight or rhodamine phalloidin (UE, Chicago, USA) incubated at room temperature in the dark for 20 min to detect F-actin. After washing, the cells were incubated with secondary antibodies at room temperature for 1 h followed by counterstaining with DAPI. The secondary antibodies were Alexa Fluor 488 goat anti-rabbit IgG (Proteintech, Chicago, USA) and Fluor 647 goat anti-mouse IgG (Fcmacs, Nanjing, China). Images were captured by confocal microscopy (Zeiss, Analytik Jena, Germany).

### Chromatin immunoprecipitation (ChIP)

ChIP assay was performed using a ChIP Kit (Abcam) following the manufacturer’s protocol. Briefly, the cells were fixed with formaldehyde to generate DNA–protein cross-links. Then, the cell lysates were sonicated to shear the DNA into fragments between 200 and 1000 bps and immunoprecipitated with H3K27ac antibody (Abcam) or a negative control IgG antibody. Precipitated DNA was purified and analyzed by real-time PCR.

### Statistical analysis

Statistical analysis was performed using SPSS 20.0 (Abbott Laboratories, IL, USA). All data were presented as the mean ± SD. Student’s *t*-test was used to analyze data between two groups. The Chi-Square test was used to examine the clinicopathological characteristics between Timeless high- and Timeless low-expressing patients. Overall survival curves were plotted using the Kaplan-Meier method and analyzed by the log-rank test. The Student’s *t*-test was used to examine the relationship between Timeless levels and clinicopathological characteristics. *P* < 0.05 was considered statistically significant.

## Results

### Timeless is upregulated in CRC tissues and associated with poor prognosis

**Fig. 1:**
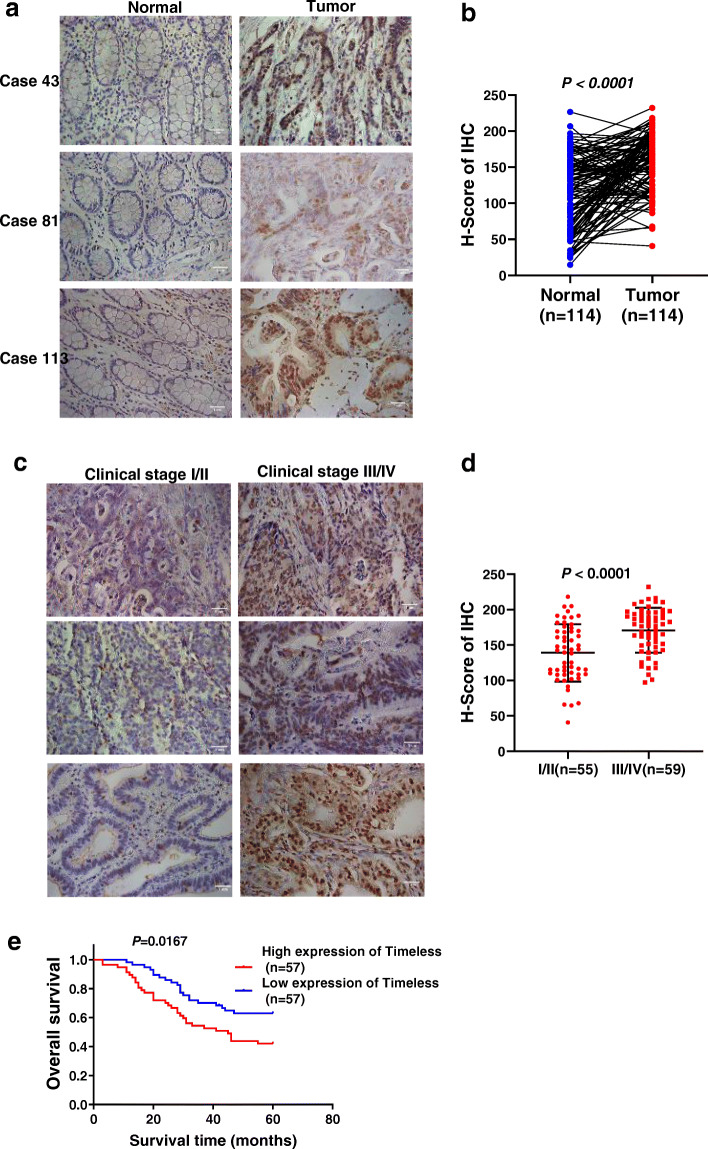
Timeless is upregulated in CRC tissues and associated with poor prognosis. (**a**) Representative images from the immunohistochemical analyses of Timeless protein expression in normal colorectal tissues and colorectal cancer tissues. (**b**) Statistical analyses of the H-score of Timeless staining in CRC and corresponding normal tissues. (**c**) Representative images from immunohistochemical analyses of Timeless protein expression in CRC tissues at different clinical stages. (**d**) Statistical analyses of the H-score of Timeless staining in CRC tissues at different clinical stages. (**e**) Kaplan–Meier survival curves showing an association between Timeless and the overall survival in patients with CRC

To investigate the role of Timeless in CRC, we analyzed the expression of Timeless protein in 114 pairs of paraffin-embedded, archived CRC specimens by immunohistochemical staining. As shown in Fig. 1a, Timeless was expressed mainly in the nucleus and rarely in the cytoplasm. The level of Timeless expression in CRC tissues was significantly increased, compared with that in corresponding adjacent normal mucosa (Fig. 1b). Timeless expression increased with the progression of CRC clinical stage (Fig. 1c, d). Moreover, patients with a higher level of Timeless had a shorter overall survival (Fig. 1e). Correlation analysis between Timeless expression and clinical pathological features verified that Timeless upregulation was significantly correlated with age (*P* = 0.021), clinical stage (*P* < 0.0001), T stage (*P* = 0.014), N stage (*P* < 0.0001), and vascular invasion (*P* = 0.005) (Table 1). However, no significant correlations were observed between Timeless expression and gender, tumor diameter, tumor location, and perineural invasion (Table 1). Collectively, Timeless was highly expressed in CRC and correlated with a poor outcome in patients with CRC.

### Timeless promotes cell proliferation and invasion in vitro

**Fig. 2:**
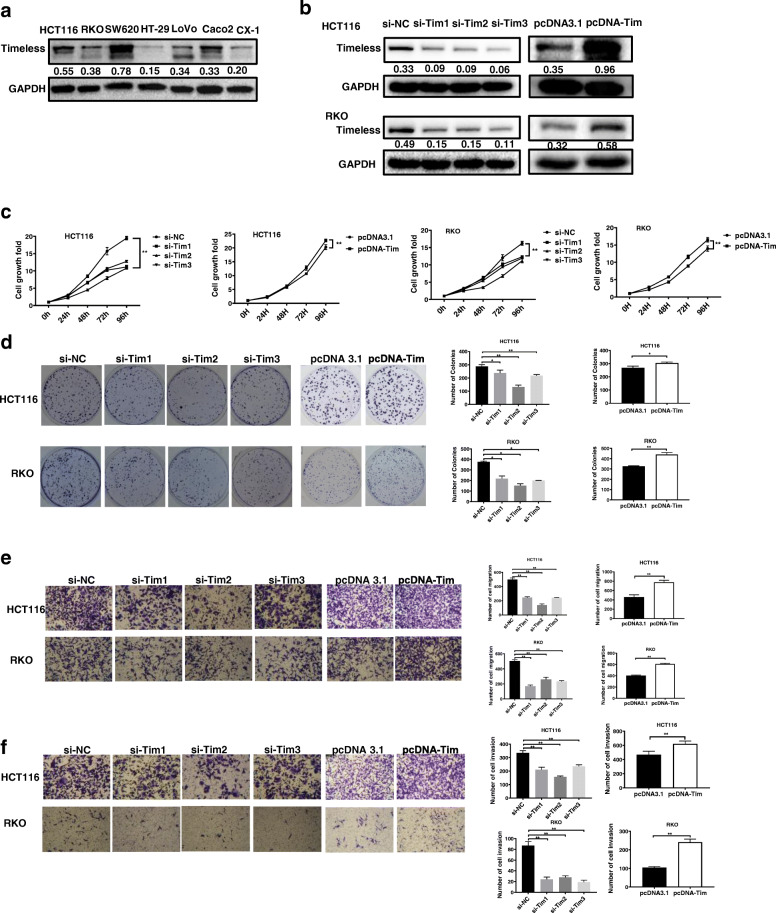
Timeless promotes cell proliferation and invasion in vitro. (**a**) The expression levels of Timeless in seven CRC cell lines were analyzed by western blotting. (**b**) Timeless expression was detected by Western blotting in HCT116 and RKO cells transfected with the indicated siRNAs or pcDNA-Tim. (**c**) CCK-8 assay and (**d**) colony-forming growth assays were performed using HCT116 and RKO cells transfected with Timeless siRNAs or pcDNA-Tim. Transwell assays were performed to detect the migration (**e**) and invasion (**f**) of Timeless-knockdown and overexpressing cells after 36–48 h. Data represent the mean ± standard deviation from three independent experiments. *P < 0.05, **P < 0.01

We next investigated the functions of Timeless in CRC cells. We measured Timeless levels in several CRC cell lines (Fig. 2a) and selected HCT116 and RKO cell lines with a medium expression of Timeless for further studies. We transiently transfected specific siRNAs and Timeless overexpressed plasmid (pcDNA-Tim) into HCT116 and RKO cells. As shown in Fig. 2b, siRNAs specifically decreased the expression of Timeless whereas pcDNA-Tim transfection increased the expression of Timeless. The results of CCK-8 assays revealed that silencing Timeless significantly suppressed the proliferation of HCT116 and RKO cells. In contrast, the overexpression of Timeless significantly increased cell viability (*P* < 0.001; Fig. 2c). Colony formation assays revealed that the knockdown of Timeless significantly inhibited the proliferation of HCT116 and RKO cell lines, whereas Timelessoverexpression cells showed an opposite effect (Fig. 2d). In transwell assays, siRNA-treated cells also showed a significant decrease in migrative and invasive potential, compared with control cells. However, Timeless-overexpressed CRC cells showed enhanced migration and invasion compared with control cells (*P* < 0.01; Fig. 2e, f).

### Timeless promotes the EMT of CRC cells

**Fig. 3:**
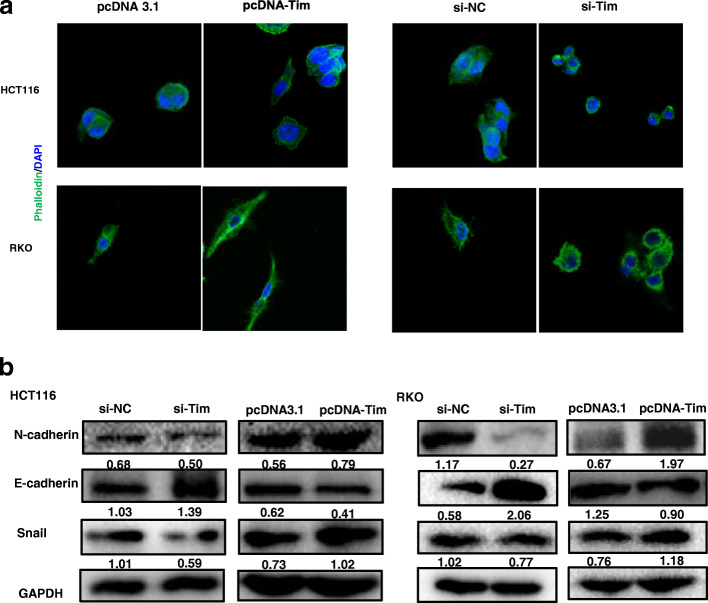
Timeless promotes the EMT of CRC cells. (**a**) Cytoskeleton detected by Rhodamine phalloidin staining in Timeless overexpressing or knockdown HCT116 and RKO cells. Original magnification, × 630 (**b**) Expression of EMT related molecules detected by western blotting in Timeless overexpressing or knockdown HCT116 and RKO cells

During these experiments, we found that the cell shapes changed upon Timeless expression. Therefore, we stained F-actin using immunofluorescence labeling with phalloidin. The cells with Timeless overexpression exhibited spindle shapes and rearrangement of F-actin, confirming to the typical morphological changes during epithelial–mesenchymal transition (EMT) (Fig. 3a) The cells with Timeless knockdown showed an opposite trend. Next, we examined whether Timeless played a role in EMT. We investigated the expression of markers associated with EMT in different Timeless-expressing CRC cells. Western blotting showed that the knockdown of Timeless significantly downregulated the mesenchymal marker N-cadherin and the transcription factor Snail, and upregulated the epithelial marker E-cadherin (Fig. 3b). Conversely, the overexpression of Timeless significantly downregulated E-cadherin and upregulated N-cadherin and Snail (Fig. 3b). Taken together, Timeless promoted the EMT in CRC cells in vitro.

### Timeless promotes the proliferation and metastasis of CRC cells in vivo

**Fig. 4:**
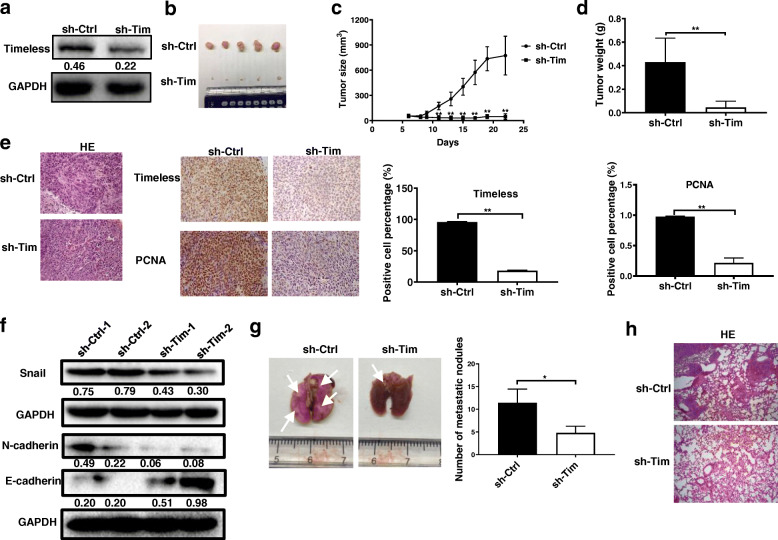
Timeless accelerates the growth and metastasis of CRC cells in vivo. (**a**) Expression of Timeless was detected in Timeless-knockdown stable HCT116 cells by western blotting. (**b**) Images of xenograft tumors from groups of BALB/c-nude mice 4 weeks after the subcutaneous injection of stable Timeless-knockdown HCT116 cells (sh-Tim) or control HCT116 cells (sh-Ctrl). (**c**) Tumor size was calculated using the equation volume = (length × width^2^)/2 every 2–3 days 1 week after injection. (**d**) Weights of xenograft tumors were measured. (**e**) Hematoxylin and eosin staining and immunohistochemistry for Timeless and PCNA in xenograft tumors. Original magnification, ×200. (**f**) Western blotting analysis of EMT-related Snail, E-cadherin, and N-cadherin in xenograft tumors. (**g**) Photos of tumor metastasis in lung tissues (left panel). The number of metastatic foci in lung tissues is quantified in the right. The white arrows show the metastases. (**h**) Hematoxylin and eosin staining of lung tissues. Original magnification, ×100. Error bars indicate the means ± SD. *P < 0.05, **P < 0.01

To determine the effect of Timeless on tumorigenesis in vivo, HCT116 cells (sh-Tim) with stable Timeless knockdown and control HCT116 cells (sh-Ctrl) were generated (Fig. 4a). Stable sh-Tim and sh-Ctrl cells were injected subcutaneously into the flanks of 5-week-old male nude mice. As shown in Fig. 4b-d, the growth, volume, and weight of tumor xenografts were significantly decreased in the sh-Tim group compared with the control group. The results of immunohistology showed that the mean expression level of Timeless in xenograft tumors from the sh-Tim group were lower than that from the control group (Fig. 4e). Likewise, expression of the proliferation marker PCNA was significantly decreased in tumors from the sh-Tim group compared with tumors from the control group (Fig. 4e). Consistent with our previous findings, decreased N-cadherin and Snail and increased E-cadherin expression levels were shown in the Timeless-knockdown sh-Tim group (Fig. 4f).

We then examined the effects of Timeless on CRC metastasis in vivo, control or Timeless-knockdown HCT116 cells were injected into nude mice via the tail vein. The results revealed that Timeless-knockdown significantly decreased colorectal cancer pulmonary metastasis (Fig. 4g). Further, H&E staining revealed that Timeless-knockdown HCT116 cells formed less nodules in the lungs comparing to control HCT116 cells (Fig. 4h).

Collectively, the knockdown of Timeless repressed the proliferation and metastasis of CRC in vivo.

### Timeless activates Wnt/ß-catenin signaling

**Fig. 5:**
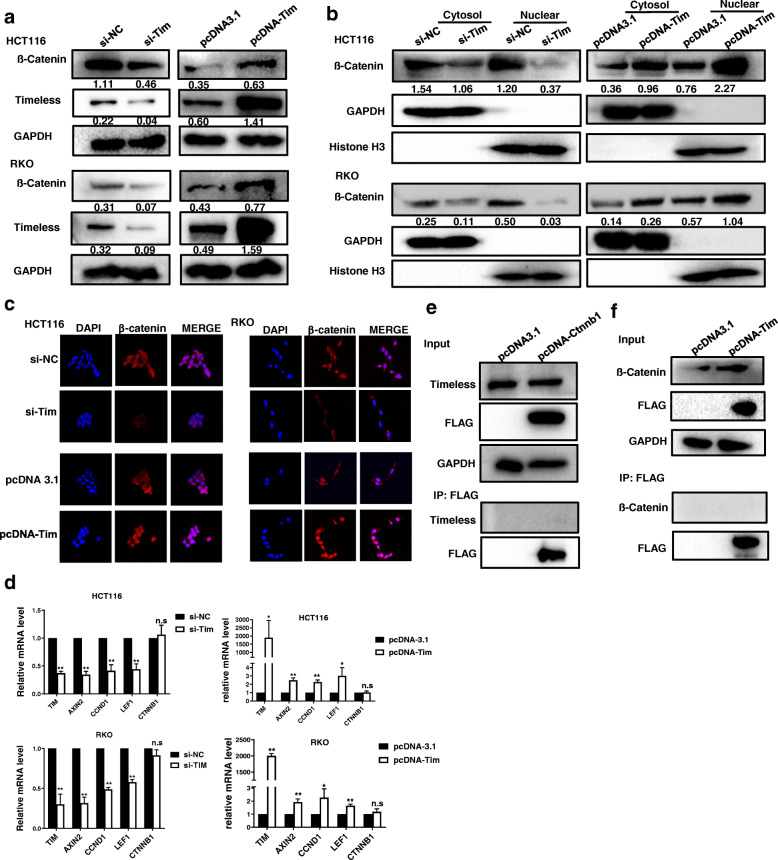
Timeless activates Wnt/β-catenin signaling. Western blotting was performed to detect total (**a**) and nuclear β-catenin (**b**) in Timeless knockdown and overexpressing CRC cells. (**c**) The laser scanning confocal microscope was performed to detect the subcellular location of β-catenin. Original magnification, × 400 (**d**) Expression levels of Axin2, CCND1, LEF1 and CTNNB1 were assayed by qRT-PCR in the indicated cells. An immunoprecipitation (IP) assay was performed to detect Flag-tagged β-catenin (**e**) or (**f**) Flag-tagged Timeless that interacted with Timeless or β-catenin in HCT116 cells. Error bars indicate the means ± SD. *P < 0.05, **P < 0.01

Previous studies have reported that the Wnt/β-catenin pathway is involved in Timeless-induced chemoresistance and EMT [22]. To investigate whether the Wnt/β-catenin pathway was involved in CRC tumorigenesis mediated by Timeless, we measured the expression of β-catenin in CRC cells with Timeless knockdown and Timeless overexpression. Upon Timeless knockdown, the levels of cellular β-catenin and nuclear β-catenin were reduced in CRC cell lines, whereas Timeless overexpression increased cellular β-catenin and nuclear β-catenin levels (Fig. 5a, b). The results from the laser scanning confocal microscope also showed high expressed Timeless induced the nuclear location of β-catenin in CRC cells (Fig. 5c). To investigate whether Timeless activated the Wnt/β-catenin pathway, we examined the expressions of genes targeted by the Wnt/β-catenin pathway in CRC cells. As shown in Fig. 5d, the expressions of Cyclin D1, Axin2, and LEF1 were markedly reduced or increased by Timeless knockdown or Timeless overexpression, respectively. However, the mRNA level of β-catenin (CTNNB1) was not affected by the expression of Timeless (Fig. 5d).

Given that Timeless participates in regulatory pathways by binding to proteins, we investigated whether Timeless interacted with β-catenin. Flag-tagged β-catenin was used to immunoprecipitate Timeless; however, no bands specific to Timeless were observed with an anti-Timeless antibody (Fig. 5e). Next, we performed an IP assay to determine whether endogenous β-catenin interacted with Timeless and found no bands specific for β-catenin (Fig. 5f). There was no interaction between Timeless and β-catenin, indicating other molecules might mediate the regulation of Timeless on β-catenin.

### Timeless binds to Myosin-9 to maintain its stability

**Fig. 6:**
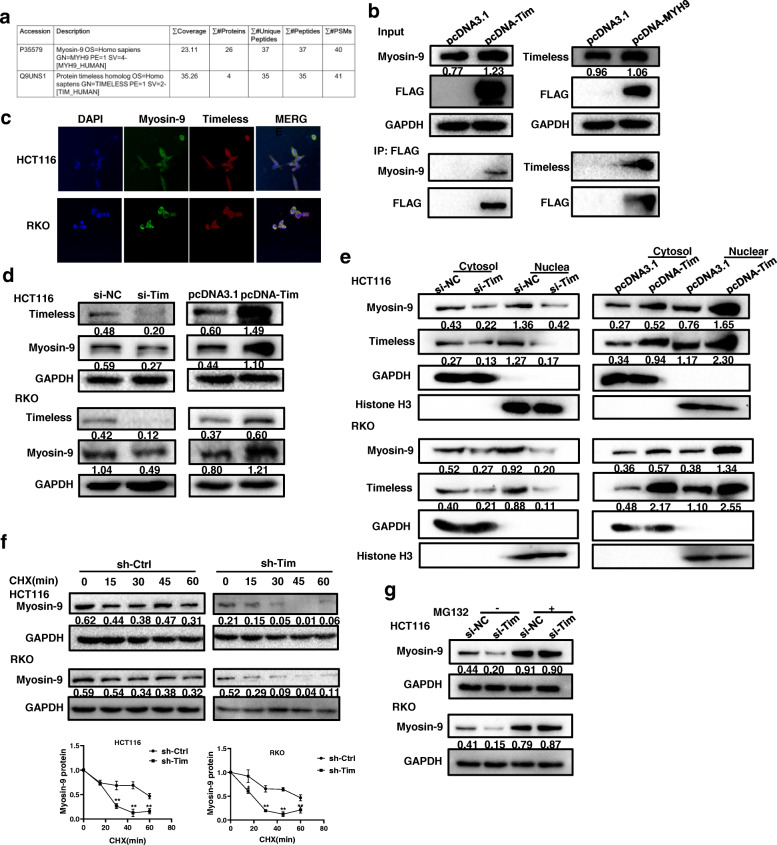
Timeless binds to Myosin-9 to maintain its stability. (**a**) Myosin-9 protein was identified by mass spectrometry. (**b**) Immunoprecipitation analyses of the interaction between Timeless and Myosin-9 in HCT116 cells. (**c**) Immunofluorescence costaining of Timeless (Red) and Myosin-9 (Green) to indicate colocalization in HCT116 (upper) and RKO (lower) cells. Original magnification, × 400. Western blotting was performed to detect total (**d**) and subcellular Myosin-9 (**e**) in Timeless knockdown and overexpressing CRC cells. GAPDH and Histone-3 were used as internal loading controls, respectively. (**f**) CRC cells with specific Timeless siRNA or control siRNA were treated with 100 μg/ml CHX and harvested at different time points as indicated. Myosin-9 was detected by western blotting and quantified by densitometry. (**g**) Timeless knockdown and control CRC cells were treated with 5 μM of MG132 for 12 h, and Myosin-9 protein was detected by western blotting. Error bars indicate the means ± SD. *P < 0.05, **P < 0.01

To identify the unknown binding proteins of Timeless, proteins were extracted from lysates of HCT116 cells and immunoprecipitated with a specific Timeless antibody. The immunoprecipitate was analyzed by mass spectrometry. Among the identified proteins, myosin heavy chain (Myosin-9) was selected for verification because of its good mass spectrometric data (Fig. 6a) and its association with CRC [26]. We transfected Flag-Timeless or Flag-Myosin-9 into HCT116 cells and extracted cell lysates for immunoprecipitation with anti-Flag and immunoblotting with anti-Myosin-9 or anti-Timeless. IP analysis showed endogenous and exogenous Myosin-9 could bind to Timeless (Fig. 6b); therefore, we detected the colocalization of Timeless and Myosin-9 in CRC cells by immunofluorescence (Fig. 6c). Timeless knockdown decreased the expression of Myosin-9, whereas Timeless overexpression upregulated Myosin-9 levels (Fig. 6d). Nucleoplasm isolation showed Timeless knockdown decreased both the nuclear and cytoplasmic levels of Myosin-9, whereas Timeless-overexpression showed an opposite effect (Fig. 6e). To test whether the binding of Timeless to Myosin-9 affected Myosin-9 stability, CHX treatment was performed, and the results showed that Myosin-9 stability was decreased by Timeless knockdown in CRC cells (Fig. 6f). MG132 treatment rescued Myosin-9 protein expression (Fig. 6g) indicating Timeless might affect Myosin-9 protein stability. Taken together, Timeless bound to Myosin-9 and increased its stability by a proteasome-dependent mechanism.

### Timeless activates β-catenin through the Myosin-9/β-catenin pathway

**Fig. 7:**
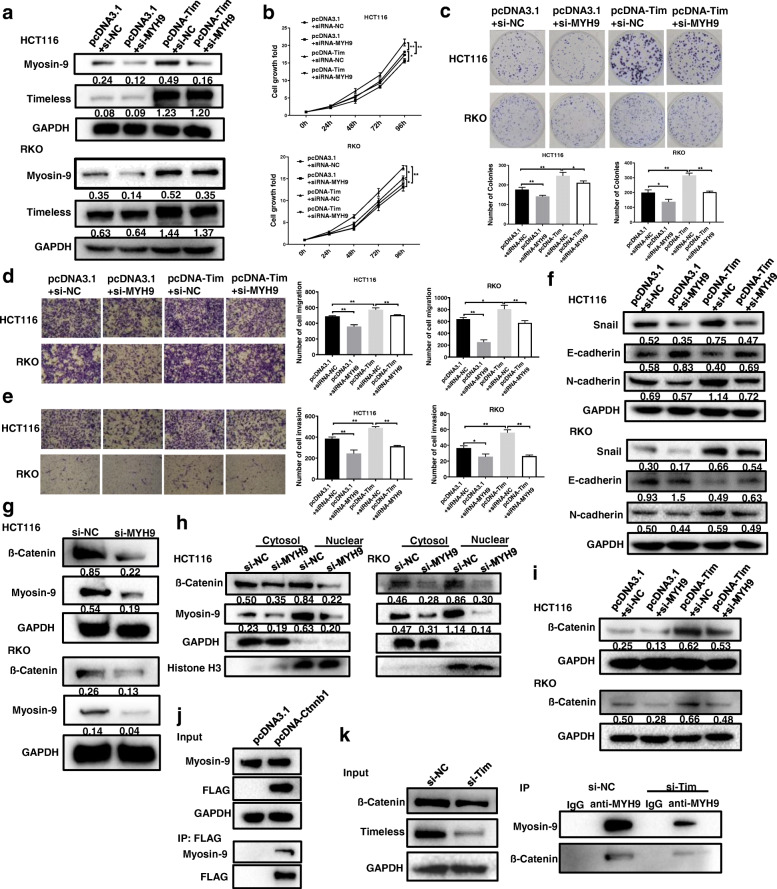
Timeless activates β-catenin through the Myosin-9/β-catenin pathway. Timeless overexpressing HCT116 and RKO cells were transfected with Myosin-9 specific si-MYH9 (pcDNA-Tim + si-MYH9) or control si-NC (pcDNA-Tim + si-NC). (**a**) Expression levels of Timeless and Myosin-9 in HCT116 and RKO cells were measured by western blotting. CCK8 (**b**) and colony-forming growth assays (**c**) were used to determine the proliferation rate of the indicated CRC cells. Transwell assays were performed to test the migration (**d**) and invasion (**e**) of the indicated CRC cells. (**f**) EMT-associated proteins in the indicated CRC cells were measured by western blotting. Expression level of total β-catenin (**g**) and subcellular β-catenin (**h**) in Myosin-9 knockdown cells were detected with Western blot. (**i**) Expression level of β-catenin was detected in CRC cells with Timeless overexpression and Myosin-9 knockdown. (**j**) Immunoprecipitation (IP) assay was performed to detect interactions between Myosin-9 and β-catenin. (**k**) Immunoprecipitation (IP) with anti-Myosin-9 was performed to detect the level of β-catenin in the cells with Timeless knockdown. Error bars indicate the means ± SD. *P < 0.05, **P < 0.01

To investigate whether Myosin-9 mediated Timeless-induced CRC cell proliferation and invasion, we knocked down Myosin-9 expression in CRC cells with Timeless overexpression (Fig. 7a). Then, we performed CCK8 and colony-forming assays, and found Myosin-9 knock-down rescued the growth of Timeless-overexpressed CRC cells (Fig. 7b, c). Transwell assay showed that a low expression of Myosin-9 retarded the migration and invasion of Timeless-overexpressed CRC cells (Fig. 7d, e). Furthermore, Myosin-9 knockdown rescued changes in the expressions of EMT molecular markers, including E-cadherin, N-cadherin, and Snail (Fig. 7f). Taken together, Myosin-9 mediated the Timeless-induced proliferation, invasion, and EMT of CRC cells.

A previous study has reported that Myosin-9 controlls β-catenin transcriptional activity by interacting with β-catenin to promote the proliferation, migration, invasion, and sphere formation of pancreatic cancer cells in vitro and in vivo [27]. We investigated whether Myosin-9 mediated the Timeless-induced activation of β-catenin. We found that Myosin-9 knockdown reduced the expression of cellular (Fig. 7g) and nuclear β-catenin (Fig. 7h). Meanwhile, Myosin-9 knockdown rescued the impact of Timeless overexpression on β-catenin expression (Fig. 7i). In addition, IP assay demonstrated that β-catenin was immunoprecipitated with Myosin-9, indicating they interacted in HCT116 cells (Fig. 7j). And this interaction was decreased upon Timeless knockdown (Fig. 7k), indicating that Timeless is required to promote Myosin-9 /β-catenin interactions. Therefore, Myosin-9 may medicate the Timeless-induced β-catenin pathway. Taken together, Timeless activated the β-catenin signaling pathway by binding to Myosin-9, which bound to β-catenin and induced its nuclear translocation.

### Timeless is transcriptionally activated by H3K27 acetylation

**Fig. 8:**
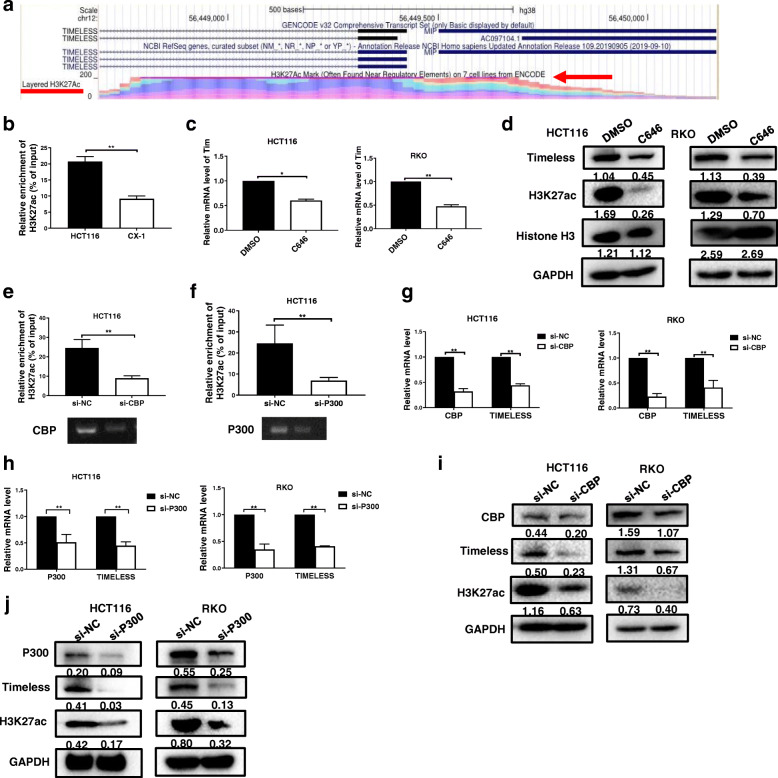
Timeless is transcriptionally activated by H3K27 acetylation. (**a**) Genome bioinformatics analysis showed that the Timeless promoter was highly enriched for H3K27ac.(cited from http://genome.ucsc.edu/) (**b**) Chromatin immunoprecipitation (ChIP) was performed to measure the H3K27ac enrichment in HCT116 cells with high a Timeless level and CX-1 cells with a low Timeless level. Timeless expression was measured by RT-qPCR (**c**) and protein levels of Timeless, H3K27ac, and total Histone-3 were measured by western blotting (**d**) in HCT116 and RKO cells treated with C646 or DMSO for 48 h. The enrichment of H3K27ac in the Timeless promoter was assessed via ChIP assay in HCT116 cells silenced by CBP (**e**) and P300 (**f**). Timeless, CBP (**g**) and P300 (**h**) mRNA expressions were measured by RT-qPCR and protein levels of CBP (**i**), P300 (**j**), Timeless, and H3K27ac in HCT116 and RKO cells were measured by western blotting

**Fig. 9:**
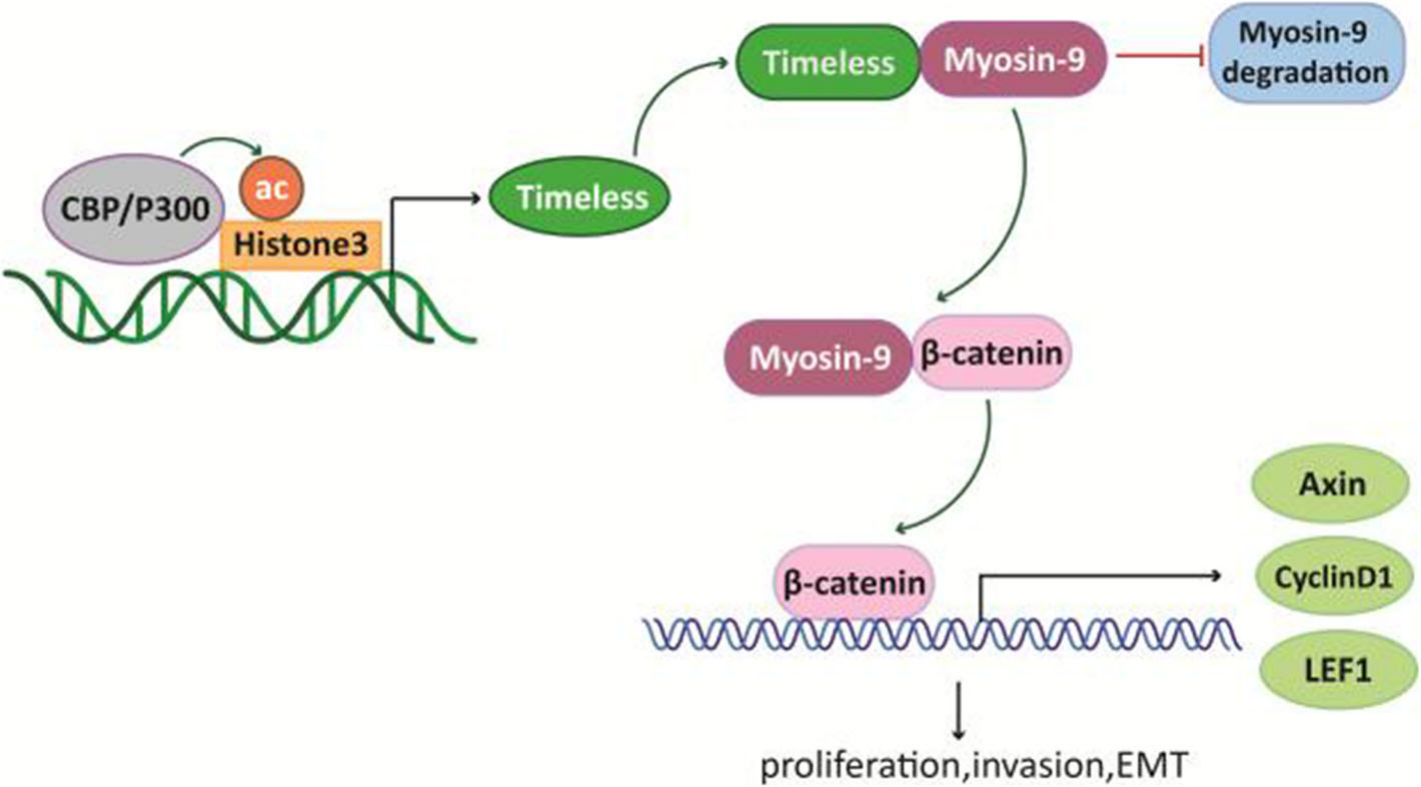
Scheme showing the proposed mechanisms involved in the effect of Timeless in CRC. H3K27 acetylation-induced Timeless bound to and stabilized Myosin-9, thereby activating the β-catenin signal pathway, which promoted colorectal cancer tumorigenesis

Histone modifications is an important epigenetic mechanism that deregulates cancer-related gene expression in tumors [28] and histone H3 lysine 27 acetylation (H3K27Ac) modification is upregulated in CRC tissues [29]. To investigate whether the increased expression of Timeless in our CRC cell lines was associated with aberrant histone modifications, we performed a genome bioinformatics analysis (http://genome.ucsc.edu/). The promoter region of Timeless was highly enriched for H3K27ac (Fig. 8a). To confirm that the increased expression of Timeless was associated with aberrant histone modifications, we performed chromatin immunoprecipitation (ChIP) to compare histone modification around the Timeless promoter in HCT116 cells (High Timeless expression) and CX-1 cells (Low Timeless expression). H3K27ac was enriched in the promoter region of Timeless in HCT116 and CX-1 CRC cells (Fig. 8b). However, the intensity of H3K27ac was markedly higher in HCT116 cells than in CX-1 cells. H3K27ac is known to be catalyzed by the P300/ CBP (CREB binding protein, CBP) complex. C646, a histone acetyltransferase (HAT) inhibitor targeting P300, significantly decreased the mRNA and protein levels of Timeless in CRC cells (Fig. 8c, d). We knocked down CBP or P300 with a specific siRNA (Fig. 8e, f) and performed ChIP with anti-H3K27ac antibody. Both CBP knockdown and P300 knockdown significantly decreased the enrichment of H3K27ac in the Timeless promoter region (Fig. 8e, f). In addition, a low expression of CBP or P300 decreased TIMELESS mRNA and protein levels in CRC cells (Fig. 8g-j). These results suggested that the upregulation of Timeless via histone acetylation in the Timeless promoter region was mediated by CBP/P300. Taken together, Timeless transcriptionally regulated by histone acetylation activated the β-catenin pathway via binding to Myosin-9 to enhance its stability. This process then promoted the proliferation, migration, invasion, and EMT of CRC cells (Fig. 9).

## Discussion

Circadian rhythm disorders may increase the risk of cancer, including colorectal cancer [4, 8]. Timeless, a core gene related to circadian clock, is upregulated in human cancers including breast, lung, colon, prostate, bladder, and pancreatic cancer [30–33]. Timeless is upregulated in human lung cancer cell lines and in clinical lung cancer tissues [31]. Furthermore, high Timeless expression is correlated with the poor overall survival of patients with lung cancer [31]. Knockdown of Timeless can suppress the self-renewal of cancer stem cells, and the invasion and migration abilities of breast cancer cells [20]. In cervical cancer, high Timeless expression is correlated with pelvic lymph node metastasis, lymphovascular space involvement, and unfavorable OS and DFS [21]. In accord with these studies, the present study found Timeless was highly expressed in CRC tissues compared with corresponding normal tissues. The Timeless expression was closely associated with clinical stage, T classification, N category, and vascular invasion. Patients with a high Timeless protein level had a worse overall survival than those with a low level. Furthermore, our functional study showed Timeless promoted the proliferation, migration, and invasion of CRC cells as well as activated EMT in vitro and in vivo.

The mechanisms of Timeless upregulation in tumors remain unclear. Previous studies have reported that the aberrant expression of Timeless in cancer is activated byERK signaling pathways [34], or by transcription factors such as E2F1 and E2F4 [35]. In addition, genetic and epigenetic regulatory mechanisms such as hypomethylation of the Timeless promoter, may mediate the expression of Timeless in breast cancer [30]. Histone acetylation modification has been proven as an important epigenetic mechanism that regulates cancer-related genes [28]. In our study, bioinformatic analysis showed the promoter region of Timeless was highly enriched for H3K27ac, and ChIP assay confirmed H3K27ac enrichment in CRC cells with high Timeless expression, compared with CRC cells with low Timeless expression. The results of histone acetyltransferase (HAT) inhibitor C646 treatment and CBP or P300 knock-down confirmed that histone acetylation regulated Timeless expression. These results suggest that the high expression of Timeless in CRC may arises from the enhancement of histone acetylation.

Timeless is mainly involved in the proliferation, apoptosis, and metastasis of tumor cells via multiple signaling pathways. Timeless boosts the progression of breast cancer through activating MYC [20]. Furthermore, Timeless confers nasopharyngeal carcinoma with cisplatin resistance by activating the Wnt/β-catenin signaling pathway and promoting epithelial mesenchymal transition [22]. Moreover, a recent study has shown that Timeless upregulation together with Claspin can protect cancer cells from oncogene-induced replication stress depending on a checkpoint-independent mechanism [36]. We also found that Timeless overexpression increased the levels of total and nuclear β-catenin and its downstream proteins, including Cyclin D1, LEF1, and Axin2. However, the knockdown of Timeless reversed these changes.

Wnt/β-catenin pathway, also termed as canonical Wnt pathway, is involved in diverse cellular processes, including embryonic development, cell proliferation, differentiation, migration, and survival [37, 38]. Hyperactivation of Wnt/β-catenin signaling is implicated in numerous cancers, including CRC [38]. The level and cellular location of β-catenin are critical in the canonical Wnt pathway [39]. Our results together with other studies, demonstrated that Timeless increased β-catenin expression and nuclear translocation to activate the Wnt/β-catenin signaling pathway.

Several studies have shown that Timeless mediates its effects by binding to other proteins such as Timeless-interacting protein (Tipin) to form a stable complex [17]. However, in the present study, we did not detect any interactions between Timeless and β-catenin, indicating that other molecules probably mediate the activation of β-catenin pathway by Timeless. To reveal the mechanism of how Timeless is involved in CRC, we performed IP with anti-Timeless antibodies and mass spectrometry followed by IP verification. We found that Timeless bound to Myosin-9, which improved its stability.

Myosin-9 encodes nonmuscle myosin IIA (NMIIA), a myosin II superfamily of motor proteins involved in important functions, including cytokinesis, cell motility, and the maintenance of cell shape. The role of Myosin-9 in cancer is controversial and depends on tumor type. Several studies have reported that Myosin-9 is an oncogene that promotes cell proliferation, invasion, and metastasis in various cancers [26, 27, 40, 41]. For example, Myosin-9 expression is significantly correlated with the depth of wall invasion, lymph node metastasis, distant metastasis, and TNM stage in gastric cancer [42]. Myosin-9 knockdown with a specific siRNA inhibits CRC cell growth and invasion [26]. In contrast, a study using a direct in vivo RNA interference (RNAi) strategy has found Myosin-9 knockdown triggers the formation of invasive squamous cell carcinoma in tumor-susceptible backgrounds by regulating p53 stability, suggesting Myosin-9 is a tumor suppressor [43]. In the present study, Myosin-9 knockdown inhibited and rescued Timeless-induced CRC proliferation, migration, invasion, EMT, and β-catenin signaling, indicating it is an oncogenic and guards the cancer-promoting effects of Timeless. Myosin-9 exerts its oncogenic effect through promoting β-catenin nuclear translocation [27]. In the present study, we showed Myosin-9 bound to β-catenin and induced its nuclear translocation, and this interaction was promoted by Timeless. In our study, Timeless did not bind to β-catenin, suggesting Myosin-9 might mediate the activation of β-catenin signaling induced by Timeless.

## Conclusion

Timeless is significantly increased in CRC tissues and is associated with advanced tumor stages and the poor overall survival of CRC patients. Timeless promots the proliferation, migration, invasion, and EMT of CRC cell via binding to Myosin-9, to induce β-catenin nuclear translocation. Timeless is transcriptionally regulated by CBP/P300-mediated histone acetylation of the Timeless promoter. Timeless may be a potential prognostic biomarker and therapeutic target for CRC.

## Data Availability

All data generated or analyzed during this study are included in this published article.

## Abbreviations

CRC: Colorectal cancer
OS: Overall survival
TNM: Tumor, node and metastasis
GAPDH: Glyceraldehyde-3-phosphate dehydrogenase
